# Eye-Level Street Greenery and Walking Behaviors of Older Adults

**DOI:** 10.3390/ijerph17176130

**Published:** 2020-08-24

**Authors:** Peng Zang, Xuhong Liu, Yabo Zhao, Hongxu Guo, Yi Lu, Charlie Q. L. Xue

**Affiliations:** 1 Department of Architecture and Urban Planning, Guangdong University of Technology,729 Dongfeng E Rd, Guangzhou 510006, China; pzang89@gdut.edu.cn (P.Z.); zhaoyb@gdut.edu.cn (Y.Z.); guohx@163.com (H.G.); 2 Department of Architecture and Civil Engineering, City University of Hong Kong, Kowloon B6322, Hong Kong; yilu24@cityu.edu.hk (Y.L.); bscqx@cityu.edu.hk (C.Q.L.X.)

**Keywords:** eye-level, street greenery, walking

## Abstract

Evidence suggests that built environment characteristics affect older adults’ travel activity behaviors, e.g., walking and cycling, which have well-established health benefits. However, the relationship between urban greenery and walking behaviors remains unclear, partly due to methodological limitation. Previous studies often measured urban greenery from a bird’s eye perspective, which may mismatch with the pedestrian’s perception from the street. In this study, we measured greenery view index from eye-level streetscape photos retrieved from Baidu Street View, an online mapping service provider. Walking behaviors of 180 older adults in six neighborhoods were collected from questionnaires. We also measured land use diversity, pedestrian-oriented design (street connectivity), and population density—the three Ds of the built environment. Results show that street greenery view index contributes to walking time of older adults, suggesting street greenery should be taken into design consideration to promote walking behaviors of older adults.

## 1. Introduction

Over the past decades, with the rise of private vehicles, urban residents in many countries have replaced active transport such as walking and cycling with car-driving. The change of travel behaviors leads to the prevalence of physical inactivity among older adults, the most sedentary segment of the population [1]. Hong Kong is currently one of the regions with the longest average life expectancy in the world, but aging has also become a major problem facing Hong Kong. The Census and Statistics Department of the Hong Kong Government announced that in 2016, the number of elderly people (elders) over 65 years old in Hong Kong reached 1163 million, an increase of 36.4% from 2006; the number of people aged 80 and over increased by 66.7% in the past 10 years.

Promoting physical activity to maintain health among older adults is an urgent need in Hong Kong, for compelling evidences that older adults greatly benefit from physical activity (such as walking),reducing chronic illness risks and improving health both physiologically and psychologically [2]. Furthermore, active transport, especially walking, constitutes the most popular habitual physical activity because it requires no special equipment or clothing and can be done alone or in the company of others at any time, making it easily incorporated into one’s daily routine [3,4]. Therefore, in the context of developing sustainable and healthy cities, encouraging walking is a key public policy priority [5]. Evidence have established that the relationship between built environment features and walking differ by the walking domain. For example, neighbourhood safety may be associated with more walking for recreation but less so for transport [5]. However, in this study, we intend to just focus on the impact of street greenery on total walking time, which helps older adults in maintaining health.

### 1.1. Built Environment and Walking

Older adults spend most of their time in neighborhoods after retiring from their work place, so their neighborhoods’ built environment is especially relevant for them. Older adults also have increased functional limitations, which lead to their fear of going outside. If the environment contains physical barriers such as long distances to destinations, slopes, and obstacles, walking behaviors may be reduced [6]. More specifically, the built environment is more likely to be associated with transportation walking compared with other types of active transport behaviors, e.g., recreational walking [7,8].

Walking is an important source of outdoor physical activity as it is easy to conduct, does not require equipment, and can be conducted anywhere, alone or with companions. One vital factor is the walkability of a neighborhood. Studies repeatedly identified three variables within the three Ds framework that support walkability: land use diversity, pedestrian-oriented design (including street connectivity), and density [9,10,11]. The three Ds framework has become a universal framework for exploring the relationship between the characteristics of the built environment and walking behavior [9,10,11,12]. Most evidence from the United States and Australia shows that walking behaviors are encouraged by a high residence and employment density, enhancing street connectivity, improving pedestrian infrastructure, and convenient facilities such as sidewalks and street trees as well as more roads to various destinations (mixed-use) [13].

However, some studies conducted in high-density cities recently in South America and Asia show that there is no significant link, or even the opposite finding, indicating that the relationship between the "Ds" and walking or physical exercise is more complex [14,15,16,17,18]. For example, a study in Hong Kong found two of these three Ds—land use diversity and street connectivity—have no significant relationship with any walking area. In addition, the third D, population density, is only positively correlated with transportation walking and leisure activities in the lower range, but negatively correlated with leisure walking in the higher range. The author believes that the relationship between the three Ds framework and walking behavior may vary in different environments. Higher density, land use diversity and street connectivity do not necessarily lead to increased walking behaviors in a densely populated city like Hong Kong [18]. So, it worth investigating whether other factors such as greenery has an effect on walking behavior, especially for the older adults.

### 1.2. Urban Greenery and Walking

Much research supports the health benefits of urban greenspaces, for they promote walking and physical activity. Kaczynski and Henderson reviewed 50 studies of parks and found in most cases that the presence of parks in a neighborhood promotes physical activity including walking [19]. However, almost all empirical studies have focused on parks and open green spaces, with very few street-level greenery and even fewer greenery-walking studies. Street-level greenery should be accessed because it considers the pedestrian and human-scale urban greenery and is more relevant to people’s daily activities. Lu reported a positive link between the quality and quantity of street greenery and recreational physical activity [20]. Both street greenery and the number of parks were associated with more walking; street greenery was associated with total walking time (excluding parks), suggesting that eye-level street greenery affected walking behavior at least as strongly as parks. Liu’s study showed a positive association between exposure to neighborhood greenness and mental wellbeing [21]. Yang also assessed street greenery using Google Street View and found positive associations with both the odds of engaging in walking and total walking time of the older adults [22].

### 1.3. Our Study

There are two major research gaps in previous studies. First, most studies measured urban greenery with a bird’s eye perspective, such as NDVI or areas of greenspaces. However, people are exposed to greenery at the eye level while walking in streets or parks. Hence, it is important to measure greenery from a pedestrian’s perspective. Second, most studies were conducted in low-density cities. The relationship between built environment characteristics and older adults’ walking behaviors remains unclear. To address these two gaps, we measured greenery view index from eye-level streetscape photos retrieved from Baidu Street View, an online mapping service provider. Big data of city street view images, such as Google or Baidu Street View, can be an easily conducted, time-saving, and economical way of measuring green exposure. Walking behaviors of 180 older adults in six neighborhoods were collected from questionnaires. We also measured the three Ds of the built environment characteristics, namely land use diversity, street connectivity, and population density. In order to implement environmental interventions to encourage older adults’ walking, we must understand how the urban greenery and other built environment affects their walking behavior in densely populated cities in Asia.

## 2. Materials and Methods 

### 2.1. Walking Data

#### Participants and Procedures

**Figure 1:**
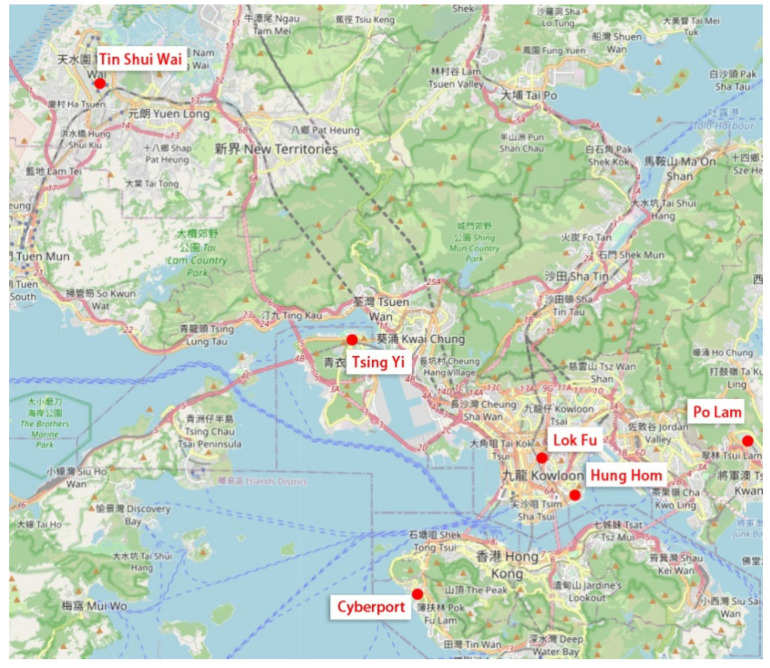
Distribution of the chosen regions in Hong Kong.

Participants were randomly interviewed within a buffer zone of 500 m in each neighborhood (using a convenience sampling method). The Chinese version of the International Physical Activity Questionnaire—Long Form was adopted, questioning participants about how much time they had spent walking in the past 7 days (see Appendix A). All participants were approached in person and recruited upon the verification of their eligibility and provision of signed informed consent. This study only focused on able-bodied people aged 65 and older; elders who needed long-term medical care in hospitals were not included. The proportion of the population aged 65 and above in these six regionsis shown in the Table 1 below:

PPS (probability proportionate to size) sampling was adopted. The response rate is approximately 50%. Since older adults usually gather in groups, one interview potentially represented at least three to four samples. Given the proportion and potential samples, only 180 samples were obtained, this is representative of the general public (Figure 1). The descriptive statistics for socio-demographic characteristics of the samples are listed in Table 2.

### 2.2. Data Analysis

In this study, neighbors are defined as living within 500 m street network buffers from participants’ residences [23]. Built environment data of land use mix and street connectivity were objectively assessed using a geographic information system (GIS) and density data from the government.

Land use mixes were calculated based on the diversity of land use with different land-use areas, which can be defined by the following equation:

*Land use mix* = −((b1/a) × ln(b1/a) + (b2/a) × ln(b2/a) + (b3/a) × ln(b3/a))/(ln(N)) (1)

where a = total areas of land for all three land uses present in the buffer zone (square meters); b1, b2, and b3 measure the areas of perspective land use (square meters); b1 represents residential areas (square meters), b2 represents commercial areas (square meters), b3 represents office areas (square meters); and *n* = the number of the three land uses with an area > 0.

A higher score represents a greater degree of mixed land use. Street connectivity is defined by the number of street intersections in a buffer. Population density data were retrieved from Census and Statistics Department of the Hong Kong government. The density was measured by the number of people per km^2^ (people/km^2^).

### 2.3. Urban Greenery

**Figure 2:**
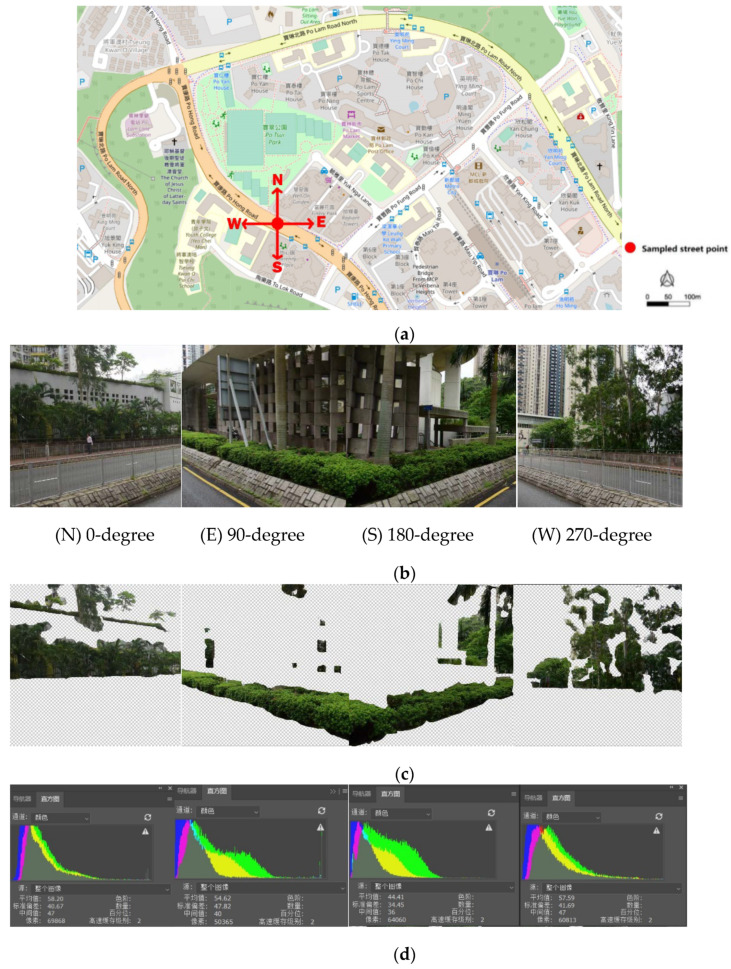
Eye-level street greenery assessment. (**a**) Sampling points with 50 m spacing were addressed in the street centerlines. (**b**) Four streetscape images were captured for each point from Baidu Street View from directions of north, east, south, and west. (**c**) All vegetation in a photo was extracted in Adobe Photoshop. (**d**) The total number of pixels representing vegetation were calculated with a histogram.

Urban greenery exposure was obtained from Baidu Street View images. The sampling points were added along streets with an interval of 50 m. We retrieved four street view images facing north, east, south, and west from each sampling point (Figure 2). Using Adobe Photoshop software (Adobe, San Jose, CA, USA), all pixels representing vegetation in a photo could be extracted and displayed in a histogram. The level of urban greenery was measured by the green view index (the ratio of green pixels in the four images), as shown in the following formula [24]:

(2) $$\mathrm{Green}\;\mathrm{view}\;\mathrm{index}=\frac{\sum_{i=1}^{4}Greenerypixels_{i}}{\sum_{i=1}^{4}Totalpixels_{i}}$$

Green view index value ranges from 0.0 to 1.0, with a higher value indicating more eye-level greenery. The average green view index of all points in the buffer zone is used to assess the communities around the place of residence. In total around 240 street view images are included in this study.

### 2.4. Other Factors

Individual information, e.g., age, gender, former occupation, and self-reported health status, was also included in the study. Variance inflation factor (VIF) for the model is 5.675.

## 3. Results

The descriptive statistics of built environment characteristics and urban greenery for six neighborhoods are shown in Table 2. The effects of sociodemographic characteristics, built environment characteristics, and urban greenery on walking time are shown in Table 3. Green view index was found to have significantly contributed to total walking time in these six regions. On the other hand, land use diversity, street connectivity, and population density were not significantly related to total walking time.

Bivariate correlation analysis was adopted in SPSS and the results are shown in Table 4. Only age and green view index have significant effects on walking time of the elderly.

## 4. Discussion

According to the latest statistics, both men and women in Hong Kong have the longest life expectancyin the world [25]. The average Hong Kong woman’s lifespan is 87–32 years, while Hong Kong men reach an average of 81–24 years of age. Healthy lifestyles, which typically involve an emphasis on walking and physical activity, may be one reason for the long lifespan in Hong Kong. However, empirical findings about the impact of the built environment on older adults’ walking behaviors in high-density cities are still limited. This study will help policymakers and urban planners to provide a better environment to promote active living for older adults in Hong Kong. By comparing different kinds of communities under the unique urban density in Hong Kong, some design guidelines and suggestions could be provided.

Urban green spaces including parks and tree-lined streets are vital to physical activities especially walking for older adults. Greenspaces can provide suitable space for walking and other physical activity. Open spaces and greenspaces are much needed in a high-density city like Hong Kong. Although Hong Kong has very high density and limited space, several characteristics contribute to the walking behaviors of older adults. Hong Kong is very compact, high-density, and has many destinations, its public transport network is well-developed and easy to access, and there is a cultural atmosphere. These factors may all contribute to much higher levels of walking observed in Hong Kong elders as compared to their Western counterparts [26,27]. Furthermore, since green view index have significant effects on walking time (shown in Table 4), urban greenery should also be taken into consideration given its impacts on walking and its scarcity in Hong Kong.

As with many other countries, Hong Kong has adopted policies of aging in place. Care for older people should ideally be set in the community, preferably allowing aging in place for as long as possible. Hence, neighborhood built and social environments should be improved to accommodate the increasing number of aging residents. Integrated planning is recommended, for it allows people to age in place with minimal necessary personal adjustments.

## 5. Conclusions

By bivariate correlation analysis in SPSS, we find only age and green view index have significant effects on walking time of the elderly, suggesting that street greenery, especially eye-level, should be taken into design consideration to promote walking behaviors of older adults.

### Limitation of the Research and Recommendation for Future Study

Several limitations exist in this research. First of all, the sample size is relatively small. In this study, only 180 elders were recruited. Further studies with a large representative sample are needed. Secondly, walking behaviors were assessed with questionnaires, which are subject to recall bias. Further studies may objectively collect such data with portable devices, such as accelerometers.

## Figures and Tables

**Table 1:** Elderly population proportion in six regions and average proportion.

	Po Lam	Hung Hom	Tsing Yi	Lok Fu	Tin Shui Wai	Cyberport
Proportion	10.9%	18%	17%	18.5%	10.8%	16.7%
Average	15.32%					

**Table 2:** Descriptive statistics for sociodemographic characteristics (*n* = 180).

Socio-Demographic Characteristics Statistics					
Gender:					
Male	102 (57%)		Female	78 (43%)	
Age:					
65–74	102 (57%)	75–84	63 (35%)	85+	15 (8%)
Education:					
No Education	30 (16.7%)	Primary	84 (46.7%)	Higher	66 (36.6%)
Former Occupation:					
Domestic	16 (8.9%)	Blue collar	117 (65%)	White collar	47 (26.1%)
Self-Reported Health:					
Poor	21 (11.7%)	Fair	83 (46.1%)	Good	76 (42.2%)
Total	180				

**Table 3:** Built environment characteristics and green view indexes of the six regions.

	Po Lam	Hung Hom	Tsing Yi	Lok Fu	Tin Shui Wai	Cyberport
Land Use Mix	0.142	0.213	0.042	0.057	0.084	0.674
Street Connectivity	99.480	107.038	118.861	71.913	92.321	101.960
Population Density	0.376	0.151	0.244	0.226	0.367	0.102
Green View Index	0.312	0.204	0.320	0.415	0.291	0.234

**Table 4:** The effects of sociodemographic characteristics, built environment characteristics, and urban greenery on walking time.

	Walking Time Beta Coefficients	*p*-value
SES characteristics		
Gender	0.09	0.22
Age	−0.20	0.01
Education	0.11	0.13
Occupation	0.04	0.63
Health	0.10	0.19
Built Environment		
Land Use Mix	0.09	0.28
Street Connectivity	−0.09	0.26
Population Density	0.14	0.10
Green View Index	0.137	0.05

## Appendix A

**Table A1:** Socio-demographic Characteristics.

Gender:	M/F		
Age:	65–74	75–84	85+
Education:	No education	Primary	Higher
Former occupation:	Household	Blue collar	White collar
Self-reported health:	Poor	Fair	Good

Please think about the time you spent walking in the last seven days, including transport walking (e.g. travel from place to place) and any other walking that you might do for recreation, exercise, leisure, etc.

**Table A2:** Physical activity.

During the last seven days, how many days did you spend at least 10 min on walking at a time? ________ Days		How much time did you usually spend walking on those days? ________ Minutes
